# Comparative analysis of ethical standards for the utilization of Assisted Reproductive Technologies in Brazil: Resolutions from 1992 to 2023

**DOI:** 10.5935/1518-0557.20240069

**Published:** 2025

**Authors:** Wanderlucia Arcelino Guedes, Laisa Kimberly Rodrigues Santos, Victoria Nascimento Milanez, Marília Lima de Brito, Gabriel Acácio de Moura, Paula Bruno Monteiro

**Affiliations:** 1 Christus University Center (Unichristus), Fortaleza, CE, Brazil; 2 Conceptus Center for Assisted Human Reproduction, Fortaleza, CE, Brazil; 3 Oswaldo Cruz Foundation (FIOCRUZ), Eusébio, CE, Brazil

**Keywords:** Assisted reproduction, ethical, resolution

## Abstract

Due to its documented records and technological achievements, in vitro embryo creation technology is still honored worldwide forty years after Louise Brown’s birth on July 25, 1978. The World Health Organization (WHO) reports that one in six people globally who are of reproductive age may have infertility at some point in their lives. In this environment, it has become noteworthy for couples or patients to want to become pregnant through operations carried out by Assisted Human Reproduction Centers (AHRCs). This continual quest for AHRCs has already been demonstrated in Brazil, where data from the National Embryo Production System (SISEMBRIO) show that as of 2022, there were around 192 clinics nationally that offered AHRC services, and a total of 284,210 frozen embryos. The ethical conundrums that these techniques provide persist notwithstanding their remarkable success in helping patients achieve clinical pregnancies and viable embryos. The absence of legislation on reproductive assistance is another significant factor pertaining to Brazilian regulatory resolutions. As a result, clinics, hospitals, and sperm banks operating in this field are required to adhere to guidelines created by the Federal Council of Medicine (CFM). The first resolution was published on September 30, 1992. In light of the aforementioned, acquiring and keeping an eye on the standards that the CFM has developed over time helps enhance knowledge of the moral and legal framework that governs Brazil. Thus, the current study attempts to provide a comparative analysis of Brazilian ethical norms regarding the use of assisted human reproduction technology.

## INTRODUCTION

Due to its documented records and technological achievements, in vitro embryo creation technology is still honored worldwide forty years after Louise Brown’s birth on July 25, 1978 (Lui Yovich, 2020). This phenomenon is directly related to the increasing number of cases of infertility. The World Health Organization (WHO) reports that one in six people globally who are of reproductive age may have infertility at some point in their lives (WHO, 2023). In this environment, it has become noteworthy for couples or patients to want to become pregnant through operations carried out by Assisted Human Reproduction Centers (AHRCs) (Sciorio & El Hajj, 2022).

This continual quest for AHRCs has already been demonstrated in Brazil, where data from the National Embryo Production System (SISEMBRIO) show that as of 2022, there were around 192 clinics nationally that offered AHRC services, and a total of 284,210 frozen embryos (SISEMBRIO, 2022). The ethical conundrums that these techniques provide persist notwithstanding their remarkable success in helping patients achieve clinical pregnancies and viable embryos (Piersanti *et al*., 2021). Consequently, over time, ongoing modifications to regulatory resolutions have been noted (Cabar et al., 2022).

The absence of legislation on reproductive assistance is another significant factor pertaining to Brazilian regulatory resolutions. As a result, clinics, hospitals, and sperm banks operating in this field are required to adhere to guidelines created by the Federal Council of Medicine (CFM), with the most recent update being released on September 20, 2022. The first resolution was published on September 30, 1992 (Costa, 2007). In light of the aforementioned, acquiring and keeping an eye on the standards that the CFM has developed over time helps enhance knowledge of the moral and legal framework that governs Brazil (Cabar *et al*., 2022). Thus, the current study attempts to provide a comparative analysis of Brazilian ethical norms regarding the use of assisted human reproduction technology.

## MATERIALS AND METHODS

This study examines regulations that the Federal Council of Medicine (CFM) released in 1992, 2010, 2013, 2015, 2017, 2021, and 2022 through a cross-sectional qualitative analysis. From August 1, 2022, to August 1, 2023, the CFM’s virtual library was used as the major source for data compilation. Three authors (GWA, SLKR, and MNV) assessed all collected data and normative directions first, while subject-matter specialists BML, MPB, and MGA analyzed the results later.

After a thorough data analysis, the following relevant themes were found and categorized: (1) General Principles, (2) Target Patients, (3) Donation of Gametes and Embryos, (4) Cryopreservation of Gametes and Embryos, (5) Disposal of Surplus Embryos, (6) Preimplantation Genetic Testing, (7) Temporary Uterus Assignment, and finally, (8) Postmortem Reproduction, alongside other modifications of lesser significance in the assisted reproduction landscape.

## RESULTS AND DISCUSSION

### General Principles

The resolutions have changed over time in a number of ways, yet in some ways they have not changed. For example, the subject of embryonic reduction-the removal of one fetus from a multiple pregnancy-has received a lot of attention, especially when seen through a religious lens, which raises questions about abortion. As a result, the resolutions governing assisted reproduction (AR) uphold the same position, comprehension, and proscription. That is to say, it is against the law to reduce embryos from babies born through AR since the 1992 resolution. Table 1 presents all of the data.

**Table 1:** Resolutions Issued by the CFM from 1992 to 2023.

	1358/1992	1957/2010	2012/2013	2121/2015	2168/2017	2294/2021	2330/2022
**Target Audience**	References women only	All capable individuals	Mentions heterosexual, homosexual, and single individuals, preserving the possibility of objection by the doctor	Maintains	Adds transgender people and removes the doctor's right to object	Maintains	All capable individuals
**Shared Pregnancy**	Not mentioned	Not mentioned	Not mentioned	Allowed shared pregnancy in female samesex relationships where there is no infertility	Explains shared pregnancy: embryo obtained from the fertilization of an oocyte from one woman is transferred to her partner	Maintains	Maintains
**Oocyte and Embryo Transfer**	Maximum of four as per patient decision	Age distribution: 35 years (2 embryos); 36-39 years (3 embryos); 40 years (4 embryos)	Age distribution: 35 years (2 embryos); 36-39 years (3 embryos); 40 years (4 embryos)	Age distribution: 35 years (2 embryos); 36-39 years (3 embryos); 40 years (4 embryos)	Maintains	< 37 years: (2 embryos); 37 years: (3 embryos). Regardless of age: 2 euploid embryos	Maintains
**Cryopreservation and Disposal**	Cryopreservation of surplus embryos is mandatory; In cases of divorce, illness, and death, the decision on their fate is up to the patients	Maintains	Possibility of discarding cryopreserved embryos after 5 years at the patients' discretion	Maintains	Embryos frozen for 3 years can be discarded according to the patient's wish; Or Embryos abandoned for >3 years can be discarded if the contract is not fulfilled	Limited to eight embryos created in the laboratory, those not transferred fresh must be cryopreserved; Does not mention a minimum time for disposal, which is only possible with judicial authorization	The limitation of 8 laboratory-generated embryos is removed; Does not mention the need for judicial authorization or a minimum time for disposal
**Age for Pregnancy**	Not mentioned	Not mentioned	Maximum age: 50 years	Maximum age: 50 years, exceptions with authorization from the CFM	Maintains	Maintains	Maintains
**Gamete and** **Embryo Donations**	Maximum of 2 pregnancies of different sexes per donor per 1 million inhabitants	More than 2 pregnancies of different sexes per donor per 1 million inhabitants is prohibited	Includes age limit for donation: 35 years - women; 50 years - men. Includes voluntary gamete donation and shared oocyte donation	Maintains	Clarifies that the same donor can contribute to as many pregnancies as desired within the same family	Changes the age limit for donation: 37 years - women; 45 years - men	Maintains
**Confidentiality in Gamete Donation**	The donation must be anonymous and noncommercial	Maintains	Maintains	Maintains	Maintains	Confidentiality does not apply in cases of gamete donation among relatives up to the fourth degree	Maintains
**Surrogate** **Pregnancy**	The surrogate must be related up to the second degree to one of the parents	Maintains	Inclusion of samesex unions. The surrogate must be related up to the fourth degree to one of the par-ents; Inclusion of mandatory docu-mentation	Maintains	Inclusion of single individuals.	The surrogate must have at least one child; The clinic should not mediate contact between the genetic donor and the surrogate	Maintains
**Assisted Reproduc-tion and Treatment**	Not mentioned	Use for treatment of genetic or he-reditary diseases	Includes the use of assisted repro-duction techniques for genetic alter-ations diagnosis and HLA screening	Maintains	Maintains	Removed infor-mation about the embryo's sex from the genetic test report	Does not mention the issue of the embryo's sex from the previous ver-sion

Given that more than one embryo may be transferred to the uterus in an attempt to increase the likelihood of conception, multiple pregnancies are a regular side effect of AR therapies, which worries experts in the field (Leite, 2019). It is clear that modifications have been made to the resolutions meant to lower this risk. The first resolution on the subject of embryo transfer was released in 1992 and had no restrictions. The initial changes were made in 2010 and were included in the resolution’s second publication. These changes specified the maximum age at which a woman could transfer two embryos into her uterus, three embryos for women between the ages of 36 and 39, and four embryos for women 40 years of age or older. This standard was in place until 2021, when it was changed to allow women under the age of 37 to transfer up to two embryos and women over the age of 37 to transfer up to three embryos. No matter the age of the patients, for euploid embryos, up to two may be transferred. However, in terms of egg and/or embryo donation and reception, the age of the donor is always considered.

### Target Patients

The resolutions have been modified to reflect how society has changed over time. Same-sex couples were listed as target patients for assisted reproductive procedures in Resolution No. 2,013/2013 (CFM, 2013); nevertheless, this resolution enabled doctors to decline treatment for ethical reasons; this exception was eliminated in Resolution No. 2,168/2017 (CFM, 2017). This modification results from the Federal Supreme Court’s (STF) 2011 (STF, 2023) decision to recognize same-sex couples as a family unit on October 14, 2011. According to the Resolution No. 2,320/2022 (CFM, 2022), which is presently in effect, all capable people are considered patients of assisted reproduction.

Even with these modifications, same-sex and single-parent families still had trouble registering their children’s civil status when they were born after receiving AR treatments. The National Council of Justice only regulated the issuance of birth certificates for infants whose parents chose assisted reproduction in 2016 with the publishing of Provision No. 52 on March 14, 2016. Prior to its release, registration could only be completed by a judge’s order.

First noted in the fifth edition of the Resolution No. 2,168/2017 (CFM, 2017), transgender patients were left out until the Resolution No. 2,294/2021 edition (CFM, 2021). A new resolution stating that “All capable individuals who have requested the procedure and whose indication does not deviate from the limits of this resolution can be recipients of assisted reproduction techniques” has been added to the most recent update (Resolution No. 2,320/2022) (CFM, 2022), which also suppresses patient citations once more. The goal of this modification was to make the norms more inclusive rather than to isolate particular societal groups.

### Gamete and Embryo Donation

The donation of gametes and embryos since resolution 1358/1992 (CFM, 1992) follows the requirements regarding the prohibition of profit or commercial nature and maintenance of anonymity between donors and recipients, in order to facilitate the donation due to the lack of connection between the parties, except for the special case included in resolution no. 2,294/2021 (CFM, 2021), allowing donation to relatives of recipients to the fourth degree. It is important to highlight that consanguinity between donor and recipient is not permitted, and the donor cannot gestate.

Prior to Resolution No. 2,121/2015 (CFM, 2015), which only addressed up to two pregnancies of different sexes per million inhabitants without addressing the circumstance of donation to the same family, Resolution No. 2,168 from 2017 (CFM, 2017) introduced the option for a donor to contribute to as many pregnancies as desired, as long as they are within the same recipient family.

As for oocyte donation, in 2013 the option of shared oocyte donation surfaced, considering the age of the donor at the time of collection. This is because, according to Resolution No. 2,294/2021 (CFM, 2021), the maximum age for gamete donation is 45 for men and 37 for women, unless the donor has previously had their gametes cryopreserved and informed the recipient beforehand.

The attending physician was in charge of selecting oocyte donors until 2017. However, in 2021, Resolution No. 2,294 (CFM, 2021) decided that, when using a gamete or embryo bank, the users must make their own decisions. In the event of a shared donation, the attending physician will select the donor with the recipient’s consent. The 2021 resolution (CFM, 2021) further stipulated that, in order to ensure traceability, embryos created by many donors must be transferred from a single source. In addition, it stated that a medical report confirming the fitness of the parties’ physical and mental condition was required.

Resolution No. 2.013/2013 (CFM, 2013) introduced the shared donation of oocytes as a means of expanding the pool of donors. Under this approach, donors and receivers who are facing reproductive difficulties split the costs of AR procedures as well as biological material. In this instance, the patient who received the biological material helps the recipient, who needs these cells, and the donor may get help paying for the therapy in return.

### Cryopreservation of Gametes and Embryos

Gametes from cancer patients before treatment, excess embryos to be transplanted later, and those who want to delay motherhood can all be preserved by cryopreservation. It is an adjunctive method to in vitro fertilization treatment. This permits the patient, if they so choose, to utilize these gametes or embryos in the future (Barbosa *et al*., 2009).

The method has undergone a number of updates since 1992. For example, cryopreservation was mandated for all embryos by CFM Resolution No. 1,358/1992 (CFM, 1992). The Resolution No. 1,957/2010 (CFM, 2010), changed this policy, stating that only viable surplus embryos should be preserved but that these frozen embryos may not be thrown away. Resolution No. 2,121/2015 (CFM, 2015), subsequently stated that the progenitors would be the ones to determine whether these embryos were donated for research, disposed of, or kept in cryopreservation (Leite, 2019).

### Disposal of Surplus Embryos

There is an ongoing worry about what happens to extra embryos in every new publication. When a couple reaches their intended child count, gets divorced, becomes unwell, or loses a spouse, it is not uncommon for them to want to get rid of their embryos (Neves & Coelho, 2020).

The debate over the fate of frozen human embryos is preceded by the broader question of “what constitutes life?” entangled with religious, legal, and biological concepts. In regard to this issue, the CFM adopts positions similar to the ASRM (American Society for Reproductive Medicine) and other developed countries, maintaining the notion that embryos are human cells and their fate is dependent on the decisions of their patients (Leite, 2019).

Early decisions prohibited the disposal of embryos; however, this position was later changed through Resolution No. 2,013/2013 (CFM, 2013), which permitted the discarding of embryos that were cryopreserved for more than five years. This position was upheld in Resolution No. 2,121/2015 (CFM, 2015). To lessen the accumulation of frozen embryos in clinics, the period was shortened to three years in the resolution that followed, Resolution No. 2,168/2017 (CFM, 2017), for disposal at the request of the patients as well as for disposal because of patient abandonment, which resulted from nonpayment of the annual maintenance fee and lack of response from the patients (Allebrandt, 2018).

Resolution No. 2,294/2021 (CFM, 2021) stated that disposal may only occur with a judge’s approval. Resolution No. 2,320/2022 (CFM, 2022), however, quickly overturned this, removing the requirement for permission because it was assumed that the patient owned the embryonic material.

A limit of eight embryos was placed on the total number of embryos that could be created by Resolution No. 2,294/2021 (CFM, 2021) in order to prevent the needless discarding of embryos. Due to the multifactorial nature of assisted reproduction treatments, which makes their outcomes unpredictable, professionals and patients opposed the viability of deciding which protocols should be used to maintain this number, sparking a series of discussions and significant mobilization in response to this regulation. As a result, Resolution No. 2,320/2022 (CFM, 2022) was released and no longer included a cap on the total number of embryos created.

### Preimplantation Genetic Testing

Preimplantation Genetic Testing (PGT) was included in the Resolution No. 2,013/2013 (CFM, 2013) and it is still in use as of the most recent update in Resolution No. 2,330/2022 (CFM, 2022). It is a technique used to analyze embryos obtained through in vitro fertilization with the goal of identifying chromosomal and genetic alterations as well as selecting HLA-compatible embryos, in cases of a previously affected child (Pompeu & Verzeletti, 2015).

There are ethical discussions around the application of this method to identify HLA- compatible embryos. Pahl & Vieira (2022) draw attention to the parents’ duty to care for their sick child, the lack of autonomy for the child born via assisted reproduction technology to become a stem cell donor, and - as a complication - the absence of regulations in Brazil governing the use of this technique for this purpose, which has led to conflicting interpretations.

Genetic analysis makes it possible to identify chromosomal disorders like aneuploidies (de Sá *et al*., 2022), which makes it possible to identify and transfer euploid embryos. As a result, there is a decrease in the emotional strain on patients and treatment expenses as the pregnancy rate rises. Resolution No. 2,294 (CFM, 2021), which went into effect in 2021, restricted the transfer of euploid embryos to a maximum of two. Up to four embryos could have been transferred in the past, increasing the chance of multiple pregnancies.

PGT also enables the determination of the embryo’s sex, which is one reason why assisted reproduction patients may wish to utilize it. However, the use of this technique solely for sex selection is not permitted. In 2021, the CFM imposed a limitation that prevented this information from being included in the report, except in cases of sexlinked diseases or sex chromosome aneuploidies (CFM, 2021). However, in 2022, this regulation was revoked, recognizing that the embryonic material is owned by the patient. The use of the technique solely for determining the sex of the embryo can be concerning, as some patients might choose not to proceed with the process due to a desire for a specific sex (Taylor-Sands *et al*., 2023).

### Temporary Uterus Assignment

The temporary assignment of a uterus, commonly referred to as “surrogacy,” was regulated in 1992, whose primary indication was the impossibility of natural gestation of a woman, but helping either heterosexual couples, samesex couples, or individuals seeking independent reproduction. In this arrangement, a woman temporarily lends her uterus to carry the baby of an assisted reproduction patient (Britto & Morais, 2022). Over the years, resolutions have been updated to include protective regulations for the surrogate, AR patients, and the child to be born.

The use of temporary uterus assignment has enabled many families to realize what was once an impossible dream, yet it can be contentious in various aspects. According to (Mackenzie *et al*., 2020), some potential issues include the surrogate becoming attached to the child or, in cases of male same-sex couples, only the partner genetically related to the child feeling like a parent, which could impact the dynamics of parental responsibility.

Therefore, the Resolution No. 2,013/2013 (CFM, 2013) resolution introduced changes regarding the clarification of parentage, requiring a certificate of clinical and emotional suitability from the surrogate, as well as spousal approval where applicable. Later, in Resolution No. 2,330/2022 (CFM, 2022), it was stipulated that having at least one living child is a necessity.

Another significant change concerned the surrogate’s relation to the parents. In Resolution No. 1,957/2010 (CFM, 2010), the surrogate was required to be a second-degree relative of one of the parents. This was changed in Resolution No. 2,013/2013 (CFM, 2013) to up to a fourth-degree relative, and in Resolution No. 2,121/2015 (CFM, 2015), there was an option to request an exception for using a surrogate without any blood relation, subject to CFM approval, a rule that remains in effect as per the current regulations, in Resolution No. 2,320/2022 (CFM, 2022).

Brazilian law explicitly prohibits the commercialization of temporary uterus assignment, and due to the impossibility of conducting such procedures in their country of residence, couples or individuals resort to relocating to countries with permissive legislation, such as Ukraine and England. This issue gained prominence with the recent war in Ukraine, where foreign families who had contracted the service in the country faced difficulties in bringing the born children home (Allebrandt, 2018).

The concerns arise when there is coercion or objectification of women for commercial purposes, situations occurring in underdeveloped countries, or even questions regarding the parentage and citizenship of the produced children that warrant reflection (Borges Junior *et al*., 2022).

There is a variation in the practice of assisted reproduction among countries due to cultural, religious, political, and/or economic reasons. The ability of each country to legislate on the matter differently and the increasing ease of crossing borders to undergo the procedure in another country lead individuals to seek out new techniques or different regulations, thereby circumventing the restrictions imposed by their country of origin (Cidrão *et al*., 2021).

### Post-Mortem Reproduction

In *post-mortem* assisted reproduction, artificial conception is performed using the genetic material of the deceased donor, facilitated by the cryopreservation technique where the material is preserved until its use is authorized based on specific consent. This consent is obtained at the time of donation and preservation, considering the potential future use of these gametes. In this regard, there have been no changes to the resolutions since their implementation, being indirectly included in Resolution No. 1,957/2010 (CFM, 2010) and directly and specifically in Resolution No. 2,013/2013 (CFM, 2013; Ribeiro, 2017).

## CONCLUSION

Throughout the years, numerous changes have occurred in the Brazilian normative resolutions drafted by the CFM. These updates were necessitated by conditions imposed on professionals in the field, technological advances that provided better results in in vitro fertilization, increased demand from patients seeking the much-coveted fatherhood, especially new family structures. Therefore, conducting this critical analysis of demands can significantly improve the conditions established by laws to enhance assisted reproduction treatments.
